# Design and Usability of a Heart Failure mHealth System: A Pilot Study

**DOI:** 10.2196/humanfactors.6481

**Published:** 2017-03-24

**Authors:** Nagla Alnosayan, Samir Chatterjee, Ala Alluhaidan, Edward Lee, Linda Houston Feenstra

**Affiliations:** ^1^ IDEA Laboratory Center for Information Systems and Technology Claremont Graduate University Claremont, CA United States; ^2^ City of Hope Duarte, CA United States; ^3^ Loma Linda University Medical Center Loma Linda, CA United States

**Keywords:** mHealth, telehealth, heart failure, human factors engineering, self-management

## Abstract

**Background:**

Despite the advances in mobile health (mHealth) systems, little is known about patients’ and providers’ experiences using a new mHealth system design.

**Objective:**

This study aimed to understand challenges and provide design considerations for a personalized mHealth system that could effectively support heart failure (HF) patients after they transition into the home environment.

**Methods:**

Following exploratory interviews with nurses and preventive care physicians, an mHealth system was developed. Patients were asked to measure their weight, blood pressure, and blood glucose (if they had diabetes). They were also instructed to enter symptoms, view notifications, and read messages on a mobile app that we developed. A Bluetooth-enabled weight scale, blood pressure monitor, glucometer, and mobile phone was provided after an introductory orientation and training session. HF nurses used a dashboard to view daily measurements for each patient and received text and email alerts when risk was indicated. Observations of usage, cases of deterioration, readmissions, and metrics related to system usability and quality of life outcomes were used to determine overall effectiveness of the system, whereas focus group sessions with patients were conducted to elicit participants’ feedback on the system’s design.

**Results:**

A total of 8 patients with HF participated over a 6-month period. Overall, the mean users’ satisfaction with the system ranked 73%, which was above average. Quality of life improvement was 3.6. Patients and nurses used the system on a regular basis and were able to successfully identify and manage 8 health deteriorations, of which 5 were completely managed remotely. Focus groups revealed that, on one hand, the system was beneficial and helped patients with: recording and tracking readings; receiving encouragement and reassurance from nurses; spotting and solving problems; learning from past experiences; and communication. On the other hand, findings also highlighted design issues and recommendations for future systems such as the need to communicate via other media, personalize symptom questions and messages, integrate other health tracking technologies, and provide additional methods to analyze and visualize their data.

**Conclusions:**

Understanding users’ experiences provides important design considerations that could complement existing design recommendations from the literature, and, when combined with physician and nurse requirements, have the potential to yield a feasible telehealth system that is effective in supporting HF self-care. Future studies will include these guidelines and use a larger sample size to validate the outcomes.

## Introduction

### Heart Failure

According to the American Heart Association’s “Impact on Heart Failure (HF) Report,” the number of individuals with HF is estimated at 5.7 million. As the incidence of HF increases with age, over 8 million individuals are expected to have HF by 2030. Moreover, the cost of care is also projected to increase 127% from an estimated US $30.7 billion in 2012 to around US $69.7 billion in 2030 [1]. HF is one of the main reasons for hospitalization in patients’ ages 65 years and older [2]. It is also the leading cause of death for men and women in the United States [3].

Managing HF presents a challenge for patients and providers. Patients have to spend extra time and effort for self-care. The self-care process starts with monitoring, then recognizing and evaluating symptoms, and goes from treating symptoms to evaluating treatments [4]. It is complex especially when comorbidities exist. In addition, it requires behavior and lifestyle changes such as losing weight, limiting sodium consumption, and adhering to medications. Individuals are capable of behavior change when they have the motivation, ability, and appropriate triggers in place [5]. To support HF patients, the American Heart Association recommends patient education and close supervision [6].

However, with the growing number of HF cases and limited clinical resources, exacerbations that occur after the transition of care are problematic for health care providers due to increasing costs and reimbursement restrictions. Although nurse-led HF support programs exist, more effective strategies are needed to identify and support patients at-risk.

### mHealth and Health Outcomes

mHealth systems have the potential to be beneficial to both patients and providers, and studies evaluating their impact on HF health outcomes have been rising rapidly. On one hand, a recent review articulated that interventions that used automated devices to collect vitals from patients resulted in a 35% decrease in all-cause mortality and a 23% reduction in the risk of HF-related hospitalizations [7]. On the other hand, a recent multisite randomized control trial found that telehealth intervention had a significant improvement on quality of life but did not reduce readmissions [8].

The various design approaches and inconsistent findings have resulted in the need to understand what features are effective and how users’ interaction with the system impacts adherence to the HF self-care process and, consequently, health outcomes [7,8]. This study aimed to fill this gap in research by describing the design of a HF mHealth system and the users’ experiences with it. Effective features, in this context, are system design features that are useful in identifying patients at-risk of deteriorations that might require emergency hospital admissions. Users’ interaction with the system is demonstrated by observations of usage frequency and the feedback received from users regarding the systems’ role in supporting self-care, usability problems, and suggestions for future designs. This study was conducted at Loma Linda University Medical Center (LLUMC), which is an academic medical center with an International Heart Institute that provides a cardiac rehabilitation program and clinic to support patients with HF. Two HF nurses manage patients after the transition of care.

## Methods

### System Design and Build

A design science research (DSR) approach was used [9]. DSR is well established in information systems research and mainly consists of 3 iterative cycles: relevance, design, and rigor. The relevance cycle is where designers incorporate specific context requirements into the design, whereas the rigor cycle is where they draw on the literature and experiences to inform the design as well. The design cycle is where artifacts are built and evaluated to test efficacy and usefulness.

According to DSR, the relevance cycle starts when the problem, opportunity, and the acceptance criteria for evaluation are identified. This began with an exploratory open-ended interview with the director of cardiovascular health and wellness at LLUMC on October 24, 2012 and lasted for 60 min. The interview highlighted the gap in care that occurs when the patient transitions from the hospital to the home environment and the need for a solution to bridge this gap because it is leading to an increased number of readmissions. Further, preliminary needs for a mHealth system were acquired from the HF team to ensure that the system is relevant to the context, and to confirm its potential impact. The requirements included (1) providing patients with devices to measure their weight, blood pressure, and blood glucose since HF patients often had diabetes as a comorbidity, (2) communicating the patient’s measures and symptoms to providers and support the clinicians in identifying individuals that need attention the most, and (3) educating the patient about maintaining a healthy life style (eg, nutrition and exercise).

**Figure 1:**
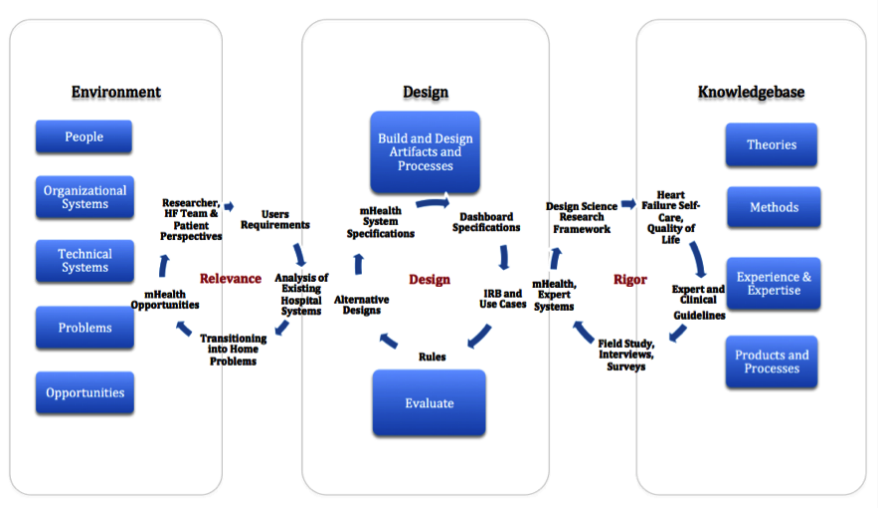
Design science research cycles, adapted from [9].

In the rigor cycle, we adapted concepts from the HF self-care theory, behavior change model, and related work to further inform the design [4,5,10,11]. Furthermore, qualitative and quantitative techniques were selected in this cycle to understand the participants’ experience and evaluate outcomes. These techniques are presented in the next section. Finally, the design cycle combined the context requirements with outputs of the rigor cycle to build the system as shown in Figure 1.

#### System Overview

**Figure 2:**
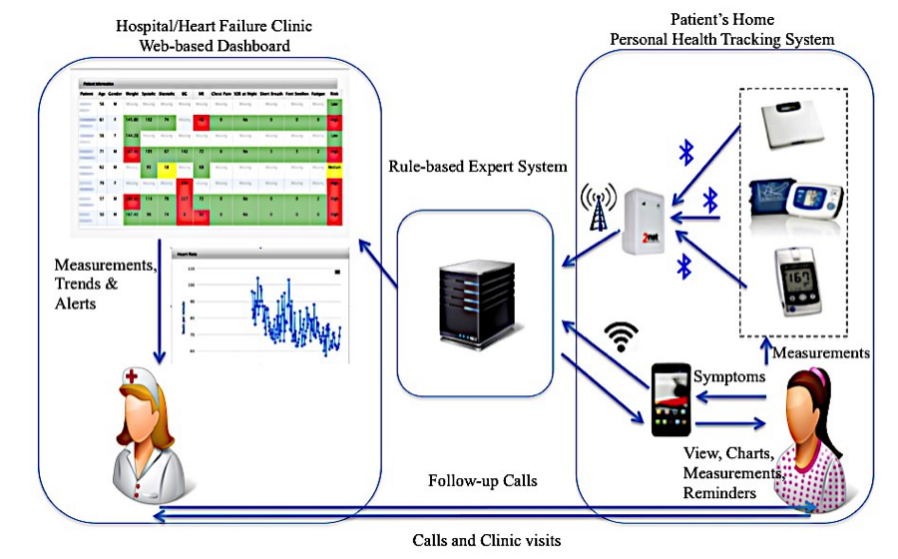
mHealth system components.

Overall, the system consisted of 3 components: (1) a personal health tracking system for each patient, (2) a rule-based expert system that collects and processes patients’ data, and (3) a dashboard view for HF nurses to view transmitted measurements. Figure 2 depicts the components and the following sections provide a brief description for each one.

##### Personal Health Tracking Component for Patients

First, the health tracking devices (Entra Health System, San Diego, CA, USA) included a wireless weight scale (A&D UC-321PBT), wireless blood pressure monitor (A&D UA-767PBT) with medium cuff, Bluetooth-enabled glucose meter with test strips and lancet supplies (MyGlucoHealth), and a hub with data service (Qualcomm 2net). Patients were also provided a mobile phone (Alcatel ONETOUCH Evolve 3G) with a custom app, called MyHeart, which was designed with continuous feedback from the HF team.

**Figure 3:**
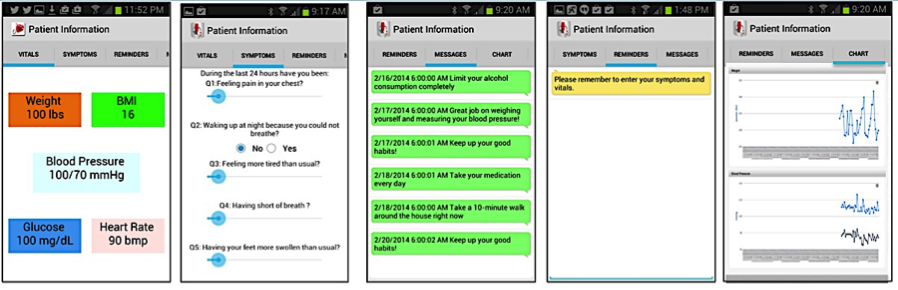
Screenshots from the mobile app.

Figure 3 shows screenshots from the mobile app. The app consisted of 6 tabs for biometrics, symptoms, reminders, messages, blood glucose, and trend charts. The symptoms section of the mobile app used a set of questions adapted from [10,12] as requested by the HF team and included the following: In the past 24 h have you been: (1) feeling chest pain? (2) waking up at night because you could not breathe? (3) feeling more tired than usual? (4) having shortness of breath? (5) having your feet more than usual? (6) feeling fatigue? A sliding scale from 0 to 10 was used for questions 1, 3, 4, and 5 because patients were already familiar with a pain scale, whereas a “yes” or “no” reply was used for questions 2 and 6 because a patient was considered at high-risk if he or she could not breathe at night.

In addition, a pool of motivational and educational messages was elicited from the preventive care physician. Examples of these messages included: “limit your total sodium intake today to no more than 1500mg,” “replace your salt shaker with fresh lemons,” “Today, make the healthy choice the easy choice.” Overall, patients received two types of notifications; reminder messages, and motivational and educational messages. Reminders were for missing data, whereas motivational and educational messages were allocated randomly and sent to patients daily.

##### Expert System Component for Data Collection and Processing

This was a cloud-based app. Rules, shown in Table 1, were practice-based and implemented to determine whether a patient was at high- or medium-risk for exacerbation that requires emergency care or hospital admission. Rules were based on the criteria used by the HF team. A patient was at high-risk if any of the measurements were in the high-risk range. Alternatively, a patient was at medium-risk if none of the measurements were in the high-risk range but at least one measurement was in the medium-risk range. This component also sent reminders and messages to patients and alerts to nurses. When data was missing for a day, the system sent a reminder notification to the patient. The nurse also made a follow-up call to determine why measurements were not received.

**Table 1:** Risk-classification rules.

Risk classification	Normal	Medium-risk	High-risk
**Measurements**			
Heart rate	60-79	50-59 80-99	49 or below 100 or above
Blood pressure systolic	90-129	80-89 130-139	79 or below 140 or above
Blood pressure diastolic	60-79	50-59 80-89	49 or below 90 or above
Weight	Gain or loss of 1 pound	Gain or loss of 1.5 pounds	Gain or loss of 2 pounds
Blood glucose	70-200	51-69 201-249	50 or below 250 or above
Symptom Q1: Feeling pain in your chest? Scale (1-10)	0-3	4-8	9-10
Symptom Q2: Waking up at night because you could not breathe? Yes or No	No	-	Yes
Symptom Q3: Feeling more tired than usual? Scale (1-10)	0-3	4-8	9-10
Symptom Q4: Having shortness of breath? Scale (1-10)	0-3	4-8	9-10
Symptom Q5: Feet more swollen than usual? Scale (1-10)	0-3	4-8	9-10
Symptom Q6: Feeling fatigue?	No		Yes

##### Dashboard View for HF Nurses

**Figure 4:**
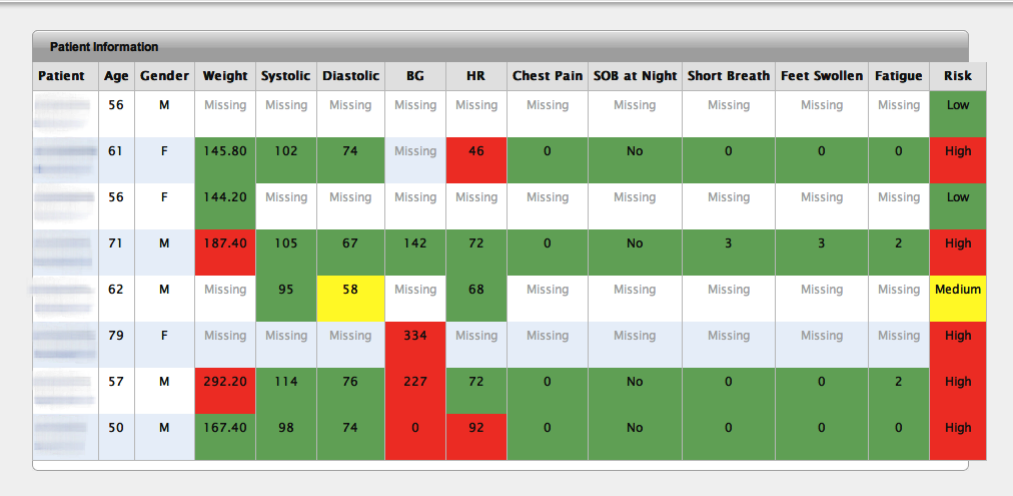
Patient list view on dashboard.

HF nurses accessed a Web-based app to view a dashboard containing patients’ data. The patient list was displayed in a table as shown in Figure 4. Each value was color-coded to indicate the status of the transmitted measurement: green for normal, orange for medium-risk, red for high-risk, and no color for missing data. Nurses also received text and email messages that alerted them when a patient was at-risk. Implementation details, including rules and security, were discussed in the previous publication [13].

### Evaluation

#### Setting and Sampling

We conducted a field study at LLUMC where the participants used the system for a 6-month period to evaluate health outcomes and usability. The HF team used purposive sampling to recruit participants from the outpatient clinic or via phone. Participants were selected to include individuals of different genders, a range of ages, socioeconomic backgrounds, and health histories. Patients were eligible if they were 21 years or older, had a clinical diagnosis of HF and one or more HF-related hospital admission in 2012, their expected survival was over 1 year, ejection fraction in the last 6 months was between 45% and 70%, and they were willing and able to use a mobile phone.

Patients were excluded if they were less than 21 years old, had comorbid conditions that may limit life expectancy to less than 1 year, unable to read text on a mobile phone due to vision disability, unable to perform self-care due to anxiety, depression, or decreased cognitive function, unable to use the monitoring equipment due to an impairment, demonstrated insufficient compliance to monitoring equipment or study visits, and had prior participation in another clinical study. Institutional Review Board (IRB) approval was obtained from LLUMC (IRB# 5130208).

In total, 12 patients were selected and invited to an orientation session at the Cardiac Rehabilitation Center conference room at LLUMC where they were informed about the purpose of this study, of which 8 patients agreed to participate and provided their consents. Reasons cited for not participating included privacy concerns.

#### Procedures

##### Observations

Two technical researchers conducted an individual hands-on 30-min training session with each patient and caregiver (if present) at the Cardiac Rehabilitation Center conference room at LLUMC. The researchers collected baseline demographics during this meeting. After that, patients returned home with their health-tracking devices, manuals, and technical support contact numbers. The measurements and usage patterns were observed daily. Hospital admissions and deteriorations were also noted when they occurred.

##### Questionnaires

Patients were asked to complete the Minnesota Living with Heart Failure Questionnaire (MLHFQ) to evaluate quality of life and the System Usability Scale (SUS) to measure satisfaction with the system. MLHFQ is a 21-item questionnaire with responses from 0 to 4. It includes 4 dimensions: global, physical, emotional, and economical. MLHFQ was selected because it has been widely used in HF studies and was accepted by the HF team as an outcome measure. The SUS questionnaire is a 10-item questionnaire with 5 response options ranging from “strongly agree” to “strongly disagree.” SUS was chosen to measure usability from each patient’s perspective because it is simple, validated, and suitable for small sample sizes. The goal was to complement other techniques, link experiences with outcome measures, and to provide additional information in a standardized format rather than to generalize the findings.

##### Focus Groups

There were 4 focus groups (3 exploratory and 1 confirmatory) held at the Cardiac Rehabilitation Center conference room at LLUMC. Focus group lasted between 1 and 2 h. The exploratory focus groups were planned to be conducted monthly to gather feedback from the patients and the HF team as they became more experienced with using the system, and to provide additional training and technical support when needed. However, due to conflicts in schedules, some group meetings were delayed but all were conducted during the study period. A confirmatory meeting, conducted at the end of the study and after saturation of concepts was reached, aimed to share the findings with participants and validate their relevance. Details for each focus group meeting are as follows:

- Focus Group A (exploratory)—2 patients, 1 nurse, 2 IT researchers—May 9, 2014
- Focus Group B (exploratory)—4 patients, 1 nurse, 2 IT researchers—May 16, 2014
- Focus Group C (exploratory)—4 patients, 3 nurses, 1 physician, 1 IT researcher—July 30, 2014
- Focus Group D (confirmatory)—4 patients, 1 nurse, 1 IT researcher—October 23, 2014

A single researcher made notes of the interviews, focus groups, and participant observations, and coded the data. Although it is recommended that two or more researchers code the data in the analysis phase and compare codes, findings were discussed with participants and other researchers to confirm validity.

Data was entered into NVivo 10 (QSR International Pty Ltd), a software package that is designed to manage unstructured qualitative data. Grounded theory coding strategies were used for analysis. Data were organized through open coding and categorization. Themes were developed and revised from emerging codes. Saturation was determined when no additional themes for users’ experiences emerged.

## Results

### Observations

#### Users

In total, 5 male and 3 female (N=8) patients with HF with a mean age of 61.5 (SD 9.3) participated in this study. It was found that 5 of the participants, 4 males and 1 female, also had type-2 diabetes. Patients also reported other health issues such as renal failure, gastroparasis, anemia, and a history of multiple heart attacks. All 8 patients were classified as stage III or IV as per the New York Heart Association (NYHA) classification. Table 2 shows patient demographics.

**Table 2:** Patient demographics.

Patient	Gender	Age in years	Has type-2 diabetes?
P1	Male	56	Yes
P2	Female	61	No
P3	Male	62	Yes
P4	Male	71	Yes
P5	Female	56	No
P6	Male	57	Yes
P7	Female	79	Yes
P8	Male	50	No

#### Usage Patterns

We observed patients use of the system on a daily basis, between March 14, 2014 and September 14, 2014, and found that they had different usage patterns. For example, 3 patients (P2, P4, and P6) used the devices consistently everyday except when camping or hospitalized, whereas 2 patients (P1 and P8) started using the system but stopped later due to changes in health providers and health coverage issues. The remaining 3 patients (P3, P5, and P7) used the devices occasionally. P5 and P7 found it challenging to remember using the system on a daily basis, whereas P3 encountered technical difficulties, with the phone connectivity and the glucose meter, that did not allow him to report blood glucose and symptom values.

**Figure 5:**
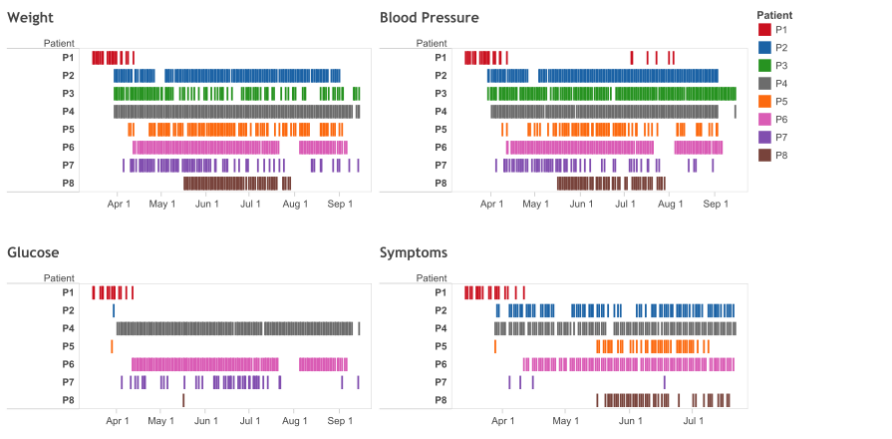
Usage patterns for weight, blood pressure, blood glucose, and symptoms.

Figure 5 shows the usage patterns for weight, blood pressure, blood glucose, and symptoms reported by the patients. Overall, automatically transmitted measurements (ie, weight, blood pressure, and blood glucose) occurred more frequently than the manually entered measurement (ie, symptoms).

### Deteriorations and Admissions

A total of 8 deteriorating cases were detected and managed. Alerts that were effective in identifying these cases were all high-risk alert events triggered by the heart rate, blood pressure, weight, and shortness of breath values. None of the medium-risk alerts were helpful in identifying patients at-risk of deterioration or admission. HF nurses were able to manage five of these conditions by calling patients and advising them to take missed medications, increase diuretics, and reduce salt intake. For example one nurse explained:

There was actually two times when it alarmed me… He’s been on the road and out and about and didn’t take his diuretic in 4 days… and I said your weight is up so much and then he dropped 8 to 10 pounds.

We also noted three HF-related admissions and no mortalities. P2 had two emergency admissions due to flu symptoms and a HF nurse guided P7 into a hospital admission when her heart rate could not be managed at home. A high-risk heart rate alerted the HF team to both patients.

### Questionnaires

#### Usability Outcomes

It was found that 6 patients 75% (6/8), who were actively using the system, responded to the SUS questionnaire. The mean SUS score was 75 (SD 17.4). A raw score of 75 converts to a percentile rank of 73% meaning that the system has higher perceived usability than 73% of all products tested [14]. This result indicates that the usability of the system was above average [15].

#### Quality of Life Outcomes

As for quality of life, 5 patients 62.5%, (5/8) who attended the first and last focus group meetings responded to the questionnaire. The baseline mean was 20.2 (SD 14.6) and the after-trial mean was 16.6 (SD 7.2). The decrease in the mean score from 20.2 to 16.6 suggests an improvement of 3.6 in quality of life. Although the overall improvement was not clinically significant because it was less than 5 [16], we noted that one patient had an improvement of 12, which was considered clinically significant.

### Focus Groups

#### Patients’ Experiences

On one hand, patients expressed that the system was useful and helped them. The following 5 themes emerged:

##### Recording and Tracking Readings

Patients stated that the system was instrumental for tracking measurements they did not track before. For instance, P4 stated that he used to track his weight and blood glucose but not his heart rate. However, he found that monitoring his heart rate helped because he had atrial fibrillation. He recalled a time when his heart rate “went wild” and he was able to watch it and call the nurse when the problem persisted. Patients also found the charts very useful and more convenient than keeping paper records.

##### Receiving Encouragement and Reassurance From Nurses

Patients were happy to receive follow-up calls from the nurse. P5, for instance, reported that the nurse monitored her measurements and called her to tell that she was doing a great job. P6 also expressed that he knew when he had a problem but the nurse called him and reassured him that he was doing what he needed to do.

##### Spotting and Solving Problems

Weight gain was often problematic for patients. P4 used the system to watch his weight to determine when fluid retention begins because he usually has shortness of breath after that. He commented: “This system of taking measurements permits me to determine when I’m building up fluid and what action I can take.”

##### Learning From Past Experiences

Patients expressed that when using the system, they started realizing how past experiences impacted their condition. For example, P1 stated that, during Easter he ate more than usual and did not pass water for days. He recalled that Easter was a time he would have been in the hospital but using the devices helped him manage his symptoms, from the “arm chair in his living room,” through laxatives and diet adjustments.

##### Communication

Patients also articulated that using the system helped them communicate with doctors and nurses especially since it was very difficult to call them directly. P2 and P5 also reported that they shared the tracked measurements with their cardiologist and that helped them make evaluations on the data. Adding a feature for the patients to text the nurse was strongly desired especially since patients sometimes knew what caused a high-risk alert and were able to manage it independently.

On the other hand, patients also pointed out areas of concerns such as reliability of the equipment and limited availability of technical support for detecting failures and resolving them immediately. Specific problems that patients experienced while using each system component are summarized along with suggested opportunities for improvement (Table 3).

**Table 3:** Usability problems and opportunities for improvement—patients’ feedback.

Component	Usability problem	Suggested opportunities for improvement in future design	Examples of patient feedback on problematic experience
Weight scale	Family members use scale	Establish validation measures for weight variations	*(The nurse) found out a problem when one of my friends used the scale and she is so tiny so the weight is 106 and then my husband used it once because he thought it’s not working and he weighs 195! So, (the nurse) was so scared and she called right away and said what happened? So, I said my friend used it and then my husband. So, it’s good. (The nurse) is the best nurse, she is always taking care of her patients*
Blood pressure monitor	Cuff usage	Include possible error in reading in training	*Sometimes you get a reading that is not a reasonable range, and, usually it is my mistake... you have to be extremely careful in how you put the cuff on...There are things that you can do wrong. You can have the tube pointed the wrong way in which point you will certainly get the wrong answer*
Blood glucose meter	Complex process to transmit readings	Allow manual entry of blood glucose values	*The blood sugar is not as easy to use as the other instruments because you have a multistep procedure to make it read properly...it is not intuitive that you first press the button to see the number visually and then you watch the countdown and then you see the thing.*
	Accuracy of readings	Test the accuracy of each device with the user before home monitoring	*The glucometer I got from you was reading me 50 points higher...well I use my own glucometer because it was a lot nicer to me in the morning because I average about 130-150 fasting*
Hub	Connectivity	Use phone as hub to verify connectivity status	*I get situations where it is not going in right...the normal procedure is to see one light blink green, the other blinking green, and then the top blinks blue. When it is red I unplug and plug-in*
App: symptoms	Redundancy in reporting symptoms everyday	Submit symptoms only when present	*It got to the point where I was just no, no, no, done…Maybe rather than having that everyday, there would be a place where we could push that symptom...if you are having it*
	Questions about symptoms do not match the patient’s personal symptom	Provide personalized symptom questions	*My symptoms aren’t the same as others... Today my eyes are drooping that is my symptom of retention of fluid so is my belly. My hands and feet are skinny*
App: messages and reminders	Not personalized	Customize messages	*I thought they were impersonal*
App: charts	Charts on the mobile app were too small and had no printing or customization capabilities	Improve chart visualizations (filtering and zooming) and provide printing and customization functions	*P2 stated that her afternoons are more problematic than her mornings and created charts on paper to show the need for a larger view and overlapping heart rate and blood pressure measurements*

#### Nurses’ Experiences

Nurses also expressed their viewpoints, which were also categorized into 5 areas:

##### Identifying Individual Patterns and Personalized Rules

Nurses highlighted that each patient had an individual pattern of measurements that require personalized rules.

##### Guiding Admission

Nurses reported that the system helped them guide admissions. One nurse described a situation when her patient’s heart rate went out of control and they were not able to manage it while she was at home, the nurse arranged her admission so the treatment was made earlier and no emergency room visit was required.

##### Recommending Treatment

Viewing daily measurements allowed nurses to recommend treatments such as taking extra diuretics.

##### Communication

Nurse-to-patient communication increased especially since the nurses contacted patients when the measurements indicated high-risk or when data were missing. They also used the charts produced from the system during the clinical visit to discuss how patients can improve their trends. The feedback, which nurses provided on each system component, is shown in Table 4.

**Table 4:** Usability problems and opportunities for improvement—nurses’ feedback.

Component	Usability problems	Suggested opportunities for improvement in future design	Example of nurse feedback
Rules	Can not customize rules to include each patient’s dry weight	Add customization capability to include dry weight	*they have to have a dry weight...having the patient know a weight that is optimal where they are not overloaded or too dry*
	Changing standards	Add customization capability to change rules according to new standards	*There are also shifts from research…we’ve been using weight gain of 2 to 3 pounds for years...they decided that is really not helpful...they are still looking at weight change but if it is 4 pounds or more...*
Dashboard: all patients	Anomalies	Allow edits or deletes	*The nurse highlighted a spike in one patient’s weight explaining that it was not real because he was weighing himself with his cat so there is a need to delete anomalies*
	Gaps in data	Improve the process to prevent gaps in data	*with it (data) not always being downloaded everyday, I see this big jump and I go what am I to do with that?*
Dashboard: individual patient views	Identifying uncontrolled cases	Control charts	*if you do a control chart, there is a pattern that starts appearing before hand and then you can see what’s going on and pay more attention*
Alerts	False positives	Add rules to reduce False Positives	*I would like to trust that text is something I need to look at… because about 95% of the time the alerts have been normal…*
			*or we wanted that to happen so we get an alert for the weight drop and we wanted the weight to drop but it comes up as a high-risk*

## Discussion

### Principal Findings

This paper presented the design and use of an mHealth system for HF. The aim was to identify what features are effective and how users’ interaction with the system impacts adherence to the HF self-care process and health outcomes. In this context, we focused on user engagement and user interaction. We explored the system usability aspects of our solution and how it benefitted both patients in achieving better health outcomes, and caregivers in providing a better way for remotely monitoring their patients. One lesson learned was that, even in spite of motivational messages and reminders, there were gaps in adherence to using the system to support self-care.

Effective features included blood pressure, heart rate, weight, and symptom monitoring as high heart rate, blood pressure, weight gain, and shortness of breath were all events that occurred and were managed immediately.

With personal health tracking features for patients and monitoring capabilities for nurses, the use of the system was correlated with an overall improvement in quality of life and detection of 8 deteriorating cases. Patients’ experiences with each system component highlighted challenges and opportunities for design improvements.

Although the HF team was a key in engaging patients to use the system, benefits that were articulated included support in recording and tracking readings, receiving encouragement and reassurance from nurses, spotting and solving problems, learning from past experiences, and communication.

In total, 3 patterns of patient usage emerged: frequent, occasional, and abandonment after initial use. These patterns emphasize the need to account for various scenarios when planning future systems to maximize the impact and reduce the cost of the system. Given that technical difficulties and remembering to take measurements were cited as reasons for occasional use, focused training, technical support, and different modes of communication could help remedy this problem. On the other hand, users who completely stopped using the system in this context, did so for two reasons: one was due to changes in health care insurance coverage which resulted in the patient becoming ineligible to continue care at the hospital. Another reason was change of health care provider as one patient transferred to another hospital because he needed a specialized service that was not offered at this setting. More research is needed to address the reasons behind system abandonment.

The following lessons were learned from the design and evaluation of the system:

#### Lesson 1: One Size Does Not Fit All

There is a need to include features that allow users to customize the mobile app and rules within the expert system for each individual case. For example, patients requested personalized messages, symptom questions, and integration with other personal medical devices and systems to overcome irrelevancy and redundancy in the mobile app. We found that some patients did not understand why they were asked about swollen feet when their swelling occurred in their stomach or eyelids. They also expressed that their expectations for messages tailored to their specific situation and lifestyle rather than a prefixed pool of messages. Some patients also articulated the need to incorporate and monitor data from other devices to track their health (eg, Implantable Cardioverter Defibrillator [ICD], Prothrombin Time and International Normalized Ratio Monitor [PT and INR], and Continuous Glucose Monitor). Nurses, on the other hand, pointed out that they need to enter a dry weight for each patient so that they can determine if the weight change is desired or not. They also requested the ability to change the rules for the expert system to keep up with changing standards.

#### Lesson 2: Visualizations Are a Valuable Feature for Patients and Nurses

Patients and HF nurses repeatedly articulated how the trend charts helped them in tracking and managing their health. Two additional features were suggested: adding control charts to help predict at-risk cases on the dashboard, and adding print and Web access capabilities for patients to customize their charts.

#### Lesson 3: Logging Mechanisms Could Be Effective When Incorporated in the Design

Patients envisioned that the ability to log known reasons behind abnormal readings on the mobile app and sending a message to inform the nurse that the problem is being treated, or is an error, would be beneficial. Nurses also preferred to have a log to indicate how each case was addressed.

#### Lesson 4: A Standard Wireless Glucose Meter for All Users Might Not Be Feasible

Although blood glucose values were a necessary requirement because HF patients could have type-2 diabetes as comorbidity, the cost of the meter and test strips supply were significant. Accuracy and reliability of the meter were also a concern. Furthermore, blood glucose values did not contribute to any of the alerts that detected deterioration.

#### Lesson 5: Information Sharing Tools and Periodic Meetings Are Desired

Patients found that group meetings helped them learn from others’ experiences and receive additional technical support. One patient suggested adding an online bulletin board to share information related to HF experiences, post announcements regarding upcoming meetings, and to discuss technical issues with the system.

### Limitations

One of the main limitations was the small sample size. However, 3-5 participants are usually considered a sufficient sample size for usability studies [17]. Furthermore, refinements to the design were not made during the study period, as this was a preliminary phase that had a restricted scope, time, and budget. Improvements will be incorporated and tested in a future study. Another limitation was, as a field study, there was a lack of control over other variables such as changes in diet and medications. As a result, the accuracy of results, such as the improvement in quality of life, could be questionable. To mitigate this limitation, we encouraged patients to discuss any negative experiences and lifestyle changes along with positive experiences.

### Comparison With Related Work

Our design complements existing research addressing the design and usability of mHealth systems. Similar to the weight and activity with blood pressure system (WANDA) in [10], we provided patients with wireless health technologies to measure and automatically send their weight, blood pressure, and blood glucose value. In terms of design, our approach confirms what has been found in [18] that an iterative approach which includes users has been shown to result in successful adoption HF telemonitoring. We also found that nurses and patients were able to spot and manage worsening cases that is consistent with [19], which found that alerts generated from transmitted blood pressure, weight, and symptoms values are effective in identifying deteriorating cases. The themes that emerged from users’ experiences, such as reassurance, confirm the benefits of using home monitoring as articulated in [11]. The lesson learned about the importance of individualized care especially for patients with comorbidities in this study supports [20] which highlighted the need to acknowledge, routinely profile, identify personal goals, support individualized case management, and include the patients’ perspective and overall outcomes in evaluation [20]. Refining a system design to be personal has also been demonstrated by Triantafyllidis et al [18].

### Conclusions

Advances in eHealth trends such as the Internet of Things are driving interest in the development and use of feasible mHealth systems. Although devices that measure health data are available to consumers, systems that allow these devices to communicate and share information with providers are needed because home monitoring could alleviate the burden of HF management on patients and providers. Features to monitor changes in weight, heart rate, and report shortness of breath could be useful for identifying deteriorating HF cases. The intelligent dashboard with automated risk classification and alerts sent to caregivers can also help to lower the burden of patient management in which typically few nurses or caregivers handle large number of cases.

We have shown that continuous monitoring infrastructure in the home can lead to better and higher quality information, which can lead to improved health outcomes as well as reduced hospital readmissions and cost savings. However, we saw the need to tailor the messages to individual preferences as important. We also saw that home logistic support is critical for widespread deployment of such technologies. We found from exit interviews that patients often are socially isolated and hence including a form of social networking technology in the app can bring them together and provide peer support. Overall, future designs should include patients’ needs such as personalized apps and messages, two-way communication with providers, enhanced visualization features, social support, and high levels of technical support. Features for providers are also needed, such as custom rules for each patient, solutions to address gaps in data, incorporation of changing standards, advanced charts, and limited alerts. Although this study did not incorporate and test these needs and is no longer used at the hospital, changes are planned for a future design.

## Abbreviations

DSR: design science research
HF: heart failure
ICD: Implantable Cardioverter Defibrillator
IRB: Institutional Review Board
LLUMC: Loma Linda University Medical Center
MLHFQ: Minnesota Living with Heart Failure Questionnaire
NYHA: New York Heart Association
PT and INR: Prothrombin Time and International Normalized Ratio Monitor
SUS: System Usability Scale
WANDA: weight and activity with blood pressure system

## References

[1] Heidenreich PA, Albert NM, Allen LA, Bluemke DA, Butler J, Fonarow GC, Ikonomidis JS, Khavjou O, Konstam MA, Maddox TM, Nichol G, Pham M, Pina IL, Trogdon JG (2013). Forecasting the impact of heart failure in the United States: a policy statement from the American Heart Association. Circ Heart Fail.

[2] Mozaffarian D, Benjamin E, Go A, Arnett D, Blaha M, Cushman M, de Ferranti S, Després J, Fullerton HJ, Howard VJ, Huffman MD, Judd SE, Kissela BM, Lackland DT, Lichtman JH, Lisabeth LD, Liu S, Mackey RH, Matchar DB, McGuire DK, Mohler ER, Moy CS, Muntner P, Mussolino ME, Nasir K, Neumar RW, Nichol G, Palaniappan L, Pandey DK, Reeves MJ, Rodriguez CJ, Sorlie PD, Stein J, Towfighi A, Turan TN, Virani SS, Willey JZ, Woo D, Yeh RW, Turner MB (2014). Heart disease and stroke statistics—2015 update. Circulation.

[3] (2016). Centers for Disease Control and Prevention.

[4] Riegel B, Lee CS, Dickson VV, Carlson B (2009). An update on the self-care of heart failure index. J Cardiovasc Nurs.

[5] Fogg B (2009). A behavior model for persuasive design.

[6] Lloyd-Jones D, Adams R, Carnethon M, De SG, Ferguson T, Flegal K, Ford E, Furie K, Go A, Greenlund K, Haase N, Hailpern S, Ho M, Howard V, Kissela B, Kittner S, Lackland D, Lisabeth L, Marelli A, McDermott M, Meigs J, Mozaffarian D, Nichol G, O'Donnell C, Roger V, Rosamond W, Sacco R, Sorlie P, Stafford R, Steinberger J, Thom T, Wasserthiel-Smoller S, Wong N, Wylie-Rosett J, Hong Y, American Heart Association Statistics CommitteeStroke Statistics Subcommittee (2009). Heart disease and stroke statistics--2009 update: a report from the American Heart Association Statistics Committee and Stroke Statistics Subcommittee. Circulation.

[7] Kitsiou S, Paré G, Jaana M (2015). Effects of home telemonitoring interventions on patients with chronic heart failure: an overview of systematic reviews. J Med Internet Res.

[8] Ong MK, Romano PS, Edgington S, Aronow HU, Auerbach AD, Black JT, De MT, Escarce JJ, Evangelista LS, Hanna B, Ganiats TG, Greenberg BH, Greenfield S, Kaplan SH, Kimchi A, Liu H, Lombardo D, Mangione CM, Sadeghi B, Sadeghi B, Sarrafzadeh M, Tong K, Fonarow GC, Better Effectiveness After Transition–Heart Failure (BEAT-HF) Research Group (2016). Effectiveness of remote patient monitoring after discharge of hospitalized patients with heart failure: the better effectiveness after transition-heart failure (BEAT-HF) randomized clinical trial. JAMA Intern Med.

[9] Hevner A, Chatterjee S (2010). Design Research in Information Systems: Theory and Practice.

[10] Suh M, Chen C, Woodbridge J, Tu MK, Kim JI, Nahapetian A, Evangelista LS, Sarrafzadeh M (2011). A remote patient monitoring system for congestive heart failure. J Med Syst.

[11] Seto E, Leonard KJ, Masino C, Cafazzo JA, Barnsley J, Ross HJ (2010). Attitudes of heart failure patients and health care providers towards mobile phone-based remote monitoring. J Med Internet Res.

[12] Jurgens C, Fain J, Riegel B (2006). Psychometric testing of the heart failure somatic awareness scale. J Cardiovasc Nurs.

[13] Alnosayan N, Lee E, Alluhaidan A, Chatterjee S, Houston-Feenstra L, Kagoda M, Dysinger W (2014). MyHeart: an intelligent mHealth home monitoring system supporting heart failure self-care.

[14] Sauro J, Lewis JR (2012). Quantifying the User Experience: Practical Statistics for User Research.

[15] Brooke J, Jordan P, Thomas B, Weerdmeester B, McClelland I (1996). SUS: A 'quick and dirty' usability scale. Usability evaluation in industry.

[16] Rector TS, Tschumperlin LK, Kubo SH, Bank AJ, Francis GS, McDonald KM, Keeler CA, Silver MA (1995). Use of the living with heart failure questionnaire to ascertain patients' perspectives on improvement in quality of life versus risk of drug-induced death. J Card Fail.

[17] Nielsen J, Landauer T (1993). A mathematical model of the finding of usability problems.

[18] Triantafyllidis A, Velardo C, Chantler T, Shah SA, Paton C, Khorshidi R, Tarassenko L, Rahimi K (2015). A personalised mobile-based home monitoring system for heart failure: the SUPPORT-HF study. Int J Med Inform.

[19] Seto E, Leonard KJ, Cafazzo JA, Barnsley J, Masino C, Ross HJ (2012). Developing healthcare rule-based expert systems: case study of a heart failure telemonitoring system. Int J Med Inform.

[20] Stewart S, Riegel B, Boyd C, Ahamed Y, Thompson DR, Burrell LM, Carrington MJ, Coats A, Granger BB, Hides J, Weintraub WS, Moser DK, Dickson VV, McDermott CJ, Keates AK, Rich MW (2016). Establishing a pragmatic framework to optimise health outcomes in heart failure and multimorbidity (ARISE-HF): a multidisciplinary position statement. Int J Cardiol.

